# Thoracic Imaging at Exacerbation of Chronic Obstructive Pulmonary Disease: A Systematic Review

**DOI:** 10.2147/COPD.S250746

**Published:** 2020-07-22

**Authors:** Bojidar A Rangelov, Alexandra L Young, Joseph Jacob, Anthony P Cahn, Sarah Lee, Frederick J Wilson, David J Hawkes, John R Hurst

**Affiliations:** 1 Centre for Medical Image Computing, Department of Medical Physics and Biomedical Engineering, University College London, London, UK; 2 Department of Computer Science, University College London, London, UK; 3 Department of Neuroimaging, Institute of Psychiatry, Psychology and Neuroscience, King’s College London, London, UK; 4 UCL Respiratory, University College London, London, UK; 5 GlaxoSmithKline Research and Development, Stevenage, UK; 6 Amallis Consulting LTD, London, UK

**Keywords:** COPD, radiology and other imaging, emphysema

## Abstract

Exacerbations of chronic obstructive pulmonary disease (COPD) are currently diagnosed based on changes in respiratory symptoms. Characterizing the imaging manifestation of exacerbations could be useful for objective diagnosis of exacerbations in the clinic and clinical trials, as well as provide a mechanism for monitoring exacerbation treatment and recovery. In this systematic review, we employed a comprehensive search across three databases (Medline, EMBASE, Web of Science) to identify studies that performed imaging of the thorax at COPD exacerbation. We included 51 from a total of 5,047 articles which met all our inclusion criteria. We used an adapted version of the Modified Newcastle-Ottawa Quality Assessment Scale for cohort studies to assess the quality of the included studies. Conclusions were weighted towards higher-quality articles. We identified a total of 36 thoracic imaging features studied at exacerbation of COPD. Studies were generally heterogeneous in their measurements and focus. Nevertheless, considering studies which performed consecutive imaging at stable state and exacerbation, which scored highest for quality, we identified salient imaging biomarkers of exacerbations. An exacerbation is characterized by airway wall and airway calibre changes, hyperinflation, pulmonary vasoconstriction and imaging features suggestive of pulmonary arterial hypertension. Most information was gained from CT studies. We present the first ever composite imaging signature of COPD exacerbations. While imaging during an exacerbation is comparatively new and not comprehensively studied, it may uncover important insights into the acute pathophysiologic changes in the cardiorespiratory system during exacerbations of COPD, providing objective confirmation of events and a biomarker of recovery and treatment response.

## Introduction

Exacerbations of chronic obstructive pulmonary disease (COPD) impose a great burden on patients’ quality of life and healthcare systems. In addition to causing significant increases in mortality and disease progression, exacerbations of COPD amount to $18 billion in direct costs annually, as well as further spending associated with care and losses in productivity.^1^ According to the Global initiative for Chronic Obstructive Lung Disease (GOLD), an exacerbation is “an episode characterized by an acute worsening of respiratory symptoms which results in additional therapy.”^2^ Reducing the frequency and severity of exacerbations is a major goal in the management of COPD.

Despite the importance of exacerbations, our understanding of these events is incomplete. In particular, the current definition of an exacerbation, being grounded solely in a change in symptoms, does not take into account underlying structural and physiological changes that occur in the lung. A worsening of respiratory symptoms may, alternatively or additionally, be caused by other co-morbid conditions. Thus, in clinical practice, exacerbation is a clinical diagnosis of exclusion – if a patient presents with worsening respiratory symptoms and if no alternative conditions are diagnosed then the patient is classified as having an exacerbation. Medical imaging may, therefore, provide the means to inform care for exacerbation in three ways. First, it could enable a “positive” diagnosis of exacerbation, which could diminish misdiagnosis due to comorbidities and facilitate quantification of the effects of novel drugs during clinical trials. Second, identifying relevant changes in the lung at exacerbation is a step towards an improved understanding of pathophysiology and might motivate the development of new therapies. Finally, discovery of rigorous imaging biomarkers of exacerbation could lay the foundations for exacerbation phenotyping.

In this work, we systematically review the available research in which thoracic imaging has been performed at exacerbation of COPD, to determine which imaging biomarkers are characteristic of an exacerbation. There is no prior systematic review of imaging biomarkers at exacerbation of COPD.

## Patients and Methods

### Study Population

We searched for studies including patients with COPD experiencing an exacerbation during which imaging of the thorax was performed. Studies that discussed imaging features that predict or correlate with future exacerbation risk but did not perform imaging at exacerbation were excluded. Studies that enrolled COPD patients for undifferentiated acute respiratory episodes were considered (even if there was no explicit definition of exacerbation), but only when they employed consecutive enrolment. Such studies were included in order to ensure we captured cases which were presenting with potential exacerbations. Studies which selectively enrolled for alternative conditions such as pulmonary embolism and pneumonia were excluded.

Both cross-sectional studies, which performed imaging only at exacerbation, and longitudinal studies, which performed imaging at additional time-points either before or after an exacerbation were included.

### Literature Search

The search strategy was developed in accordance with the Preferred Reporting Items for Systematic Reviews and Meta-Analyses (PRISMA) guidelines^3^ and the PICO/PECO framework^4^ with the following fields:

- Problem: Chronic Obstructive Pulmonary Disease (COPD)
- Exposure: Exacerbation of COPD
- Comparison: Stable COPD
- Outcomes: Catalogue of imaging features (“biomarkers”) which characterize an exacerbation.

We employed a broad search strategy. Three databases were searched up to November 24, 2019 – Medline, EMBASE and Web of Science. We used text terms, Medical Subject Headings (MeSH) terms in Medline and Emtree terms in EMBASE. The terms we used can be grouped into three categories: terms describing COPD, terms describing exacerbations and terms describing imaging. We matched the search strategies in each of the three databases as closely as possible. There were no filters imposed on the searches in order to maximize sensitivity.

The systematic review was registered on the International Prospective Register for Systematic Reviews – PROSPERO (Unique ID: CRD42018095417)^5^ before the start of data extraction. The type of articles included were all original research studies. We did not find any existing systematic reviews on the topic. Reviews, editorials, letters, opinions or conference abstracts and proceedings were excluded. Only articles in English and studies in human subjects were included. For the purposes of this review, imaging was defined as encompassing the typical clinical imaging modalities (CT, X-ray, MRI, Ultrasound, PET, SPECT, etc.), but not microscopy or direct cell imaging techniques.

**Figure 1:**
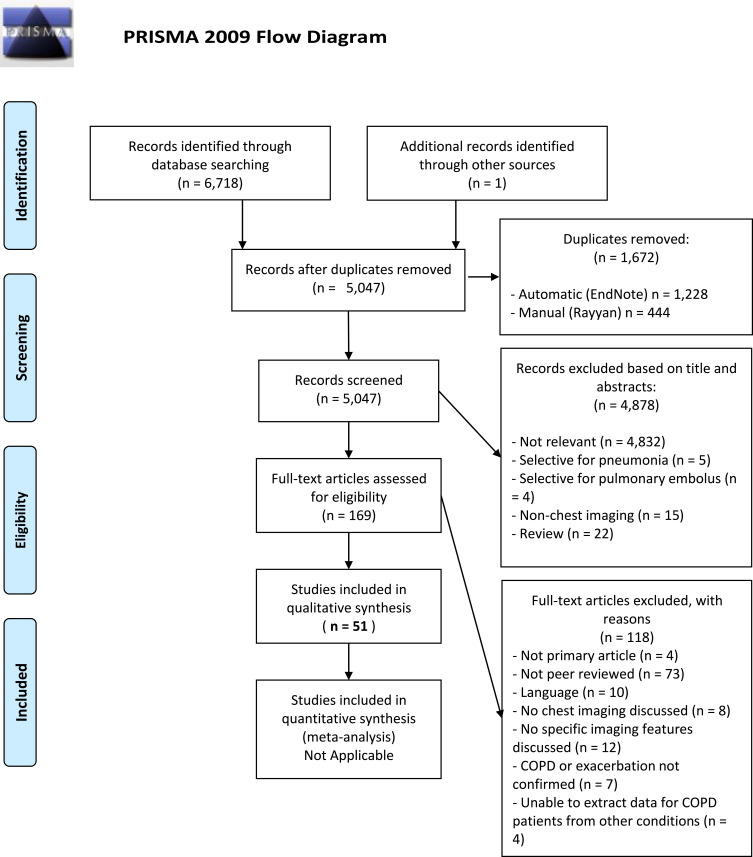
PRISMA flow chart. Studies were excluded both at screening and at full-text review, leading to a total of 51 studies included in the qualitative synthesis.

The review proceeded by first combining and de-duplicating search results in the software package EndNote, Philadelphia, PA, USA.^6^ Further to this, the de-duplicated results were exported to the online systematic review platform Rayyan, Doha, Qatar,^7^ where screening of titles and abstracts was independently performed by two authors (BAR and JRH). The authors were blinded to each other’s decisions. At this stage, further identification and removal of duplicate records was performed (Figure 1). Upon completion of screening, conflicts between the two authors were resolved by reading the complete papers and discussion. After screening, the included studies were exported to another EndNote library and classified by imaging modality. The articles were then read in full and articles that were found to not meet inclusion criteria were excluded.

### Data Extraction

A standardized data extraction table for capturing key information was developed before reading the full studies (Table 1). We also created a list of Imaging Biomarkers, included as Table S1.

**Table 1:** Imaging Features (“Biomarkers”) at Exacerbation of COPD

Paper, Year(Reference #)	Number of subjects (n =)	COPD severity when stable (mean ± SD) unless stated	Timing of imaging	Stable state imaging	Imaging features	Imaging features values	Remarks	QA Score
**Chest X-ray**								
**Alotaibi, 2018^9^**	304	FEV_1_% = 54% ± 22%for the CT and Chest X-ray patients combined	unreported	none	- Cardiac enlargement- Pleural effusion - Pulmonary congestion	- Cardiac enlargement (mild to severe enlargement) (16.2%)- Pulmonary Oedema (mild to severe) [grouped into pulmonary congestion] (15.5%)- Pleural Effusion (11.6%)	- This paper is also included in the CT section- Study correlated blood biomarkers with imaging biomarkers: blood N-terminal prohormone brain natriuretic peptide (NT-proBNP) concentrations associated with cardiac enlargement (AUC=0.72, p=0.001), pulmonary oedema (AUC =0.63, p=0.009), and pleural effusion on Chest X-ray (AUC =0.64, p=0.01)	**3**
**Emerman, 1993^43^**	254	unreported	unreported	none	- Infiltration- Masses- Pneumothorax - Pulmonary congestion	Abnormalities identified in a total 109/685 exacerbations (16%) and 19% of admitted patients, some patients having more than one abnormality:- 88 new infiltrates (13%)- 2 new lung masses (0.3%)- 1 pneumothorax (0.15%)- 20 episodes of pulmonary oedema [grouped into pulmonary congestion] (3%)	- Paper discussed a total of 685 exacerbations in 254 patients - All patients were hospitalized in the emergency department- Implied comparison to stable Chest X-ray	**1**
**Feldman, 2015^44^**	34	- GOLD I: 1 patient (3%)- GOLD II: 1 patient (3%)- GOLD III: 14 patients (41%)- GOLD IV: 3 patients (9%)- GOLD unknown: 15 patients (44%)	unreported	none	- Bronchiectasis- Bullae- Consolidation - Granuloma- Hyperinflation- Interstitial changes- Pleural effusion	- Hyperinflation in 29/33 (88%)Parenchymal changes:- Bullae in 13/33 (39%)- Consolidation in 11/33 (33%)- Bronchiectasis in 6/33 (18%)- Bronchovascular distortion in 4/33 (12%)- Cavitation in 1/33 (3%)- Granuloma in 1/33 (3%)- Interstitial changes (bilateral reticulonodular shadowing) in 4/33 (12%)- Pleural changes (pleural effusion, pleural thickening or reaction, and pleuroparenchymal bands) in 8/33 (24%)	- All patients hospitalized- Of 34 chest X-rays, 23 were reported as inadequate and 1 was deemed not-interpretable- Further detail about lobar location of parenchymal changes available	**1**
**Fuso, 1995^45^**	590	unreported	- within 5 days of exacerbation	none	- Cardiac enlargement- Inflammatory exudates - Pulmonary congestion	- Cardiac enlargement (Cardiomegaly) in 115/590 (19.5%) - Pulmonary oedema [grouped into pulmonary congestion] in 153/590 (25%)- Inflammatory exudates in 53 (9%)	- Retrospective study over 10 years- All patients were hospitalized- Chest X-ray assessed by 2 radiologists- No exclusion criteria- Pulmonary oedema and pneumonia found to be significant predictors of mortality	**1**
**Hassen, 2019^46^**	131	unreported	unreported	none	- Atelectasis - Cardiac enlargement - Pleural effusion	- Atelectasis in 6/131 (5%) patients - Cardiomegaly [Cardiac enlargement] in 16/131 (12%) patients - Pleural effusion in 23/131 (18%) patients	- Study reports findings in both chest X-ray and computed tomography - Prospective study between March 2013 and May 2017 - Contrast CT performed - All subjects hospitalized and requiring mechanical ventilation - US of lower limb also performed - Exclusion: other causes of respiratory deterioration such as pneumothorax, pneumonia, pleural effusion, pulmonary oedema, iatrogenic factor; renal failure, hypersensitivity to contrast material,taking anticoagulant therapy for any cause	**2**
**Hoiseth, 2013^47^**	99	FEV1/FVC% = 45% ±14%	- upon hospital admission	none	- Pulmonary congestion	- Pulmonary congestion in 32/195 chest X-rays (16%) and 16/99 patients (16%), based on standardized assessment	- All patients hospitalized - 41 of the patients had 1 or multiple readmissions resulting in 218 radiographs - 57 of the patients died within 1.9 years - Pulmonary congestion found to be a predictor of mortality through partial association with heart failure - Pulmonary congestion diagnosed based on the presence of one of the following: Kerley B lines, enlarged vessels in the lung apex (redistribution), peribronchial cuffing, perihilar haze, and interstitial or alveolar oedema	**2**
**Johnson, 2013^32^**	156	- GOLD 1: 5 patients (3%) - GOLD 2: 29 patients (19%) - GOLD 3: 47 patients (30%) - GOLD 4: 31 patients (20%) - No spirometry within 5 years: 44 patients (28%)	- within 24h of admission	none	- Consolidation - Pulmonary congestion	- COPD-related changes in 52/188 (28%) - Infective/inflammatory changes in 29/188 (15%) - Congestive heart failure [grouped into pulmonary congestion] in 15/188 (8%) of patients - Other (cancer, scarring, atelectasis, plaques) in 49 (26%)	- All patients hospitalized - Readmissions led to 195 total admissions with 90-day readmission rate of 44%, and 188 total radiographs - This study is an audit quantifying adherence to recommended guidelines for management of exacerbations	**3**
**Myint, 2011^39^**	9,338	Pneumonia patients:- <50%: 533/754 (71%) - 50–74%: 174/754 (23%) - ≥75%: 47/754 (6%) Non-pneumonia patients: - <50%: 3,007/4,221 (71%) - 50–74%: 960/4,221 (23%) - ≥75%: 254/4,221 (6%)	- within 24h of admission	none	- Consolidation	- Consolidation in 1,505/9,338 (16%) of patients	- All patients hospitalized - Analysis of the 2008 UK National COPD audit data- Radiological pneumonia during an AE was a predictor of worse outcomes	**1**
**Niksarlioglu, 2019^33^**	63	unreported	- upon hospital admission	None	- Bronchiectasis - Cardiac enlargement - Emphysema - Infiltration - Pleural effusion	Posterior-anterior lung radiography findings: - Bronchiectasis in 20/63 (31.7%) of patients - Cardiac enlargement (cardiomegaly) in 12/63 (19%) of patients - Emphysema in 38/63 (60.3%) of patients - Infiltration in 34/63 (54%) of patients - Pleural effusion in 17/63 (27%) of patients	- All patients ICU hospitalized with COPD exacerbation between December 1, 2011, and December 31, 2012 - Retrospective cohort study - Exclusion: lung cancer, acute respiratory distress syndrome, kyphoscoliosis, acute pulmonary embolism, acute coronary syndrome	**3**
**Saleh, 2015^38^**	14,111	- GOLD 1: 171 patients (1%) - GOLD 2: 1949 patients (14%) - GOLD 3: 3343 patients (24%) - GOLD 4: 1881 patients (13%) - No or missing spirometry: 5774 patients (41%)	- upon hospital admission	none	- Consolidation	- Consolidation in 2,714/14,111 (19%) of patients	- All patients hospitalized - Analysis of the European COPD Audit - Subjects with other radiological findings which might influence management or outcomes, including interstitial infiltrates, nodular lesions, pleural effusions or pneumothorax were excluded from the analysis - Consolidation patients admitted for exacerbation had a more severe illness	**2**
**Shafuddin, 2019^35^**	350	FEV1% = 38% ± 16%	- upon hospital admission	none	- Cardiac enlargement	- Cardiac enlargement [Cardiomegaly] in 89/350 (25%) of patients - Cardiomegaly defined as cardio-thoracic ratio of more than 0.5 in the posterior-anterior view - Cardiomegaly found as only radiographic feature with at least moderate inter-rater agreement	- All patients hospitalized - 2 prospective cohorts: one (247 subjects) from mid-July 2006 to mid-July 2007; another (176 subjects) from August 2012 to July 2013 - Chest X-rays available for only 350 patients - Exclude: patients with otherchronic respiratory diseases, pneumonia, a primary diagnosisof acute coronary syndrome or acute heart failure and those who were unable to provide written informed consent - COPD severity when stable reported for all patients (423 subjects) as impossible to extract chest radiography only- 148/423 patients had pre-existing cardiac disease	**3**
**Sherman, 1989^48^**	242	unreported	unreported	none	- Consolidation - Pneumothorax - Pulmonary congestion	Chest X-ray considered to be abnormal only when not ‘compatible to COPD’ - Chest X-ray abnormal in 35/242 (14%) - Pulmonary Oedema [grouped into pulmonary congestion] in 7 (3%) - Congestive heart failure [grouped into pulmonary congestion] in 8 (3%) - Consolidation in 3 (1%) - Pneumothorax in 1 (0%)	- All patients hospitalized - Patients split into ‘predominant clinical pattern asthma’ and ‘predominant clinical pattern emphysema/chronic bronchitis’	**2**
**Sriram, 2017^49^**	53	FEV_1_% = ~ 41% ± 18%	- within 24h of admission	none	- Infiltration - Pulmonary congestion	- Infiltration in 18/53 (33.9%) - Pulmonary congestion in 2/53 (3.8%)	- Main modality: lung ultrasound - paper appears again in the “Ultrasound and Doppler Section" - All patients hospitalized - Convenience sample - Excluded patients with renal impairment, coexisting asthma and/or bronchiectasis, acute coronary syndrome or cardiac failure - FEV_1_% value for 2 groups of patients presented in the paper: mean value approximated by author	**2**
**Titova, 2018^50^**	113	- Pneumonia subjects FEV1% = 27% (IQR 20% - 42%) - Non-pneumonia subjects FEV1% = 29% (IQR 22% - 41%)	- at admission	none	- Consolidation	- Pneumonia, defined as new infiltrate as compared to baseline radiograph [assume consolidation], in 35/113 (31%) of patients; comparison to previous baseline radiograph implied but not explicitly stated	- Prospective, single centre, observational study - Exclusion: known malignant disease, bronchiectasis, chronic bacterial colonization of the airways, treatment with immunosuppressive drug, long-term treatment with antibiotic, lack of chest X-ray	**2**
**Williams, 2018^34^**	108	- GOLD II: 45% - GOLD III: 40% - GOLD IV: 15%	- within 72h of exacerbation onset	none	- Consolidation	- Pneumonia, reported as pneumonic infiltrates [assume consolidation] in 46 of 108 (42.6%) patients with exacerbation	- Prospective, observational outpatient cohort study, patients followed monthly - Study aimed to detect new infiltrates in the lungs of exacerbation patients - Total subjects included were 127, 108 had exacerbations. The total number of exacerbations during the follow period was 355 - Exclusion: long-term antibiotic and/or CS therapy	**3**
**Computed Tomography (CT)**								
**Akpinar, 2013^21^**	148	- GOLD 2: 65 patients (44%) - GOLD 3: 38 patients (26%) - GOLD 4: 45 patients (30%)	- within 4h of admission	none	- Pulmonary embolism	- Pulmonary embolism in 56/148 (38%)	- All patients hospitalized - Prospective study with consecutive enrolment between June, 2012 and January, 2013 - Patients with deep vein thrombosis on lower extremity Doppler US, but not thrombus on CT were excluded. - Exclusion criteria: haematological diseases, coagulation disorders, hepatic or renal diseases, on oral antiplatelet or oral anti-coagulant therapy, known malignancies or collagen vascular diseases at admission - There is a probability that his study’s sample is a partial or complete subsample of the study by Akpinar, 2014 below, even though the exclusion criteria are slightly different	**1**
**Akpinar, 2014^20^**	172	- GOLD I: 12 patients (7%) - GOLD II: 64 patients (37%) - GOLD III: 49 patients (29%) - GOLD IV: 47 patients (27%)	- within 24h of admission	none	- Pulmonary embolism	- Pulmonary embolism in 50/172 (29%) Locations of PE (n (%) of all patients): - Main pulmonary artery 10/172 (5.8%) - Segmental 8/172 (4.7%) - Subsegmental 32/172 (18.6%) Sidedness of PE (n (%) of all patients) - Unilateral 45/172 (26.2%) - Bilateral 5/172 (2.9%)	- All patients hospitalized - Prospective study with consecutive enrolment between May 2011 and May 2013 - Exclusion criteria: contrast hypersensitivity, chronic renal disease, pneumonia, or congestive heart failure; anticoagulant treatment; unable to give consent because of confusion or dementia	**3**
**Alotaibi, 2018^9^**	117	FEV_1_% = 54 ± 22for the CT and chest X-ray patients combined	unreported	none	- Aortic diameter - Bronchial wall geometry - Bronchiectasis - Consolidation - Emphysema- Ground glass opacity - Interstitial disease - Mosaic attenuation - Mucous plugging - Nodules - Pulmonary Artery (PA) diameter - PA/A ratio - Pericardial effusion - Pleural effusion - Pulmonary congestion	- Aortic diameter (34.1±3.8mm)- Bronchial wall geometry (airway thickening) (67.5%)- Bronchiectasis (23.1%) - Consolidation (32.5%) - Emphysema (paraseptal) (65.8%) - Emphysema (centrilobular) (77.8%) - Emphysema (panacinar) (9.4%) - Ground glass opacity (24.8%) - Mosaic attenuation (10.3%)- Mucous plugging (49.6%) - Nodules (46.2%) - PA diameter. (28±4.7mm) - PA/A ratio (1.24±0.21) - Pericardial effusion (0.85%) - Pleural effusion (22.2%) - Pulmonary oedema (moderate to severe) [grouped into pulmonary congestion] (6%) - Reticulation [assume equivalent to interstitial disease] (4.27%)	- Paper discusses both chest X-ray (n=304, documented in the Chest X-ray section) and CT (n =117) patients- 62.4% of patients had IV contrast- Study correlated blood biomarkers with IBs: NT-proBNP associated with pleural effusion (AUC =0.71, p=0.002); serum C-reactive protein (CRP) concentration associated with pleural effusion (AUC =0.72, p=0.001), consolidation (AUC =0.75, p=0.001), ground glass opacities (AUC =0.64, p=0.028)	**3**
**Bahloul, 2015^22^**	131	unreported	- within 48h of ICU admission	none	- Pulmonary embolism	- Pulmonary embolism in 23/131 (17.5%) of patients	- Spiral CT performed only on PE suspicion (39%) - A more 'severe' sample of exacerbations as only patients admitted to ICU were considered (most had shock) - Exacerbation + PE was compared to exacerbation only; exacerbation + PE was found to be predictive of mortality	**2**
**Cheng, 2015^11^**	106	- At follow-up (n=29), pre-BD: FEV_1_% = 44.77 ± 18.54	unreported	n=16, 1-year post eCOPD	- Emphysema	- Emphysema - median LAA% based on -950 HU = 6.6, interquartile range 2.4-12.1 (n=106)At exacerbation vs follow-up (n=16): - Good LAA% correlation (r = 0.840, p <0.001) - No significant difference in LAA% (13.38% ± 9.04% vs 11.43% ± 7.1%, p = 0.135)	- %LAA > 7.5% found to be predictive of 1-year mortality	**4**
**Cheng, 2016^10^**	40	- At follow-up (n=12), FEV_1_% = 48 ± 24	unreported	Rescan in 3 months: n=12 with the same CT parameters, n=28 with routine follow up CT scans	- Bronchial wall geometry - Emphysema - Infiltration	At exacerbation vs follow-up: Bronchial wall geometry - Increase in 3rd generation WA%: 82.7±6.1% vs 79.8±5.6% (p=0.003) - Increased mean wall attenuation: 3rd gen: -215±91 vs. -283±101 HU (p<0.001) 4th gen: -312±115 vs. -382±119 HU (p=0.001) 5th gen: -414±138 vs.-463±139 HU (p=0.027) - Increased peak wall attenuation: 3rd gen: -128±105 vs. -212±111 HU (p<0.001) 4th gen: -242±130 vs.-330±133 HU (p<0.001) 5th gen: (-361±156 vs. -429±156 HU (p=0.008) - Increased lumen attenuation 3rd gen: -922±114 vs. -961±26 HU (p=0.02) 4th gen: -891±128 vs. -929±66 HU (p=0.032) 5th gen: -863±118 vs. -912±67 HU (p=0.029) - Decrease in mean inner lumen area and inner radius of airways WA% in 4th to 6th generations and wall thickness increase during exacerbation - No change in emphysema - LAA%: 9.54±6.54 vs. 9.62±6.68 (p=0.910) - No change in lung volume:- 5,482±1,038 ml vs. 5,666±985 ml (p=0.237) - No change in infiltration (61.5% prevalence)	- Patients recruited in the emergency department - Quantification with Airway Inspector Slicer 2.8 - More data available on lung infiltration patterns and distribution - in general no change was present at exacerbation vs. follow-up	**4**
**Davoodi, 2018^23^**	68	unreported	- within 72h of admission	none	- Pulmonary embolism	- Pulmonary thromboembolism in 5/68 (7.4%) of patients	- Cross-sectional study, consecutive enrolment - Exclusion: history of warfarin use, active cancer, surgery within the last two months, intolerance to contrast media - Echocardiography also performed to detect PE effects- FEV1 and FVC measured, but only at exacerbation	**2**
**Gunen, 2010^24^**	131	Available for 116/131 patients: - GOLD II: 14 patients (12%) - GOLD III: 23 patients (20%) - GOLD IV: 79 patients (68%)	- within 24h of admission	none	- Pulmonary embolism	- Pulmonary embolism in 18/131 (13.7%) of patientsLocations of PE (n (%) of all patients): - Centrally located 9/131 (7%) - Segmental 5/131 (4%) - Subsegmental 4/131 (3%)Sidedness of PE (n (%) of all patients): - Bilateral 9/131 (7%) - Right sided alone 7/131 (5%) - Left sided alone 2/131 (2%)	- All patients hospitalized - Prospective study - Consecutive inclusion - Patients with pneumothorax excluded - Presence of PE leads to a marked increase in 1-year mortality	**3**
**Hassen, 2019^46^**	131	unreported	unreported	none	- Pulmonary embolism	- Pulmonary embolism in 18/131 (13.7%) of patients - Segmental pulmonary embolism in 44% of affected patients	- Study reports findings in both chest X-ray and CT - Prospective study between March 2013 and May 2017 - Contrast CT performed - All subjects hospitalized and requiring mechanical ventilation - US of lower limb also performed - Exclusion: other causes of respiratory deterioration such as pneumothorax, pneumonia, pleural effusion, pulmonary oedema, iatrogenic factor; renal failure, hypersensitivity to contrast material, taking anticoagulant therapy for any cause	**2**
**Hackx, 2015^30^**	44	- GOLD I: 2 patients - GOLD II: 13 patients - GOLD III: 18 patients - GOLD IV: 11 patients	unreported	76 days mean interval to scan post exacerbation (minimum of 4 weeks)	- Bronchial wall geometry - Mediastinal or hilar lymphadenopathy - Pulmonary embolism	- Bronchial Wall thickening severity improves from exacerbation to follow-up: Reader 1: 14/27 patients (p<0.001); Reader 2: 12/27 patients (p=0.028) - Mediastinal or hilar lymphadenopathy improves from exacerbation to follow-up:Reader 1: 13/44 patients (p<0.001); Reader 2: 8/44 patients (p=0.008) - Low prevalence of Pulmonary Embolism at exacerbation ~6% (inter-reader agreement)	- Hospitalization required for admission to study - 2 radiologists graded images on a 4-point scale mostly based on features defined by the Fleischner Society Glossary of Terms for Thoracic Imaging - A total of 15 imaging features were graded - Values reported only when both radiologists found a statistically significant change - No exclusion criteria - Scans at exacerbation used intravenous contrast while follow up did not	**4**
**Hajian, 2018^19^**	42	- GOLD II: 17 patients - GOLD III: 19 patients - GOLD IV: 6 patients	- at exacerbation	- 6-8 weeks post exacerbation recovery	- V/Q mismatch	Ventilation-perfusion ratio (V/Q) based on imaging metrics of ventilation and perfusion iV and iQ: - iV - image-based volume at TLC minus the image-based volume at FRC - iQ - blood vessel density at TLC multiplied by image volume at TLC - Significant changes in iV/Q ratio, driven primarily by iV; numerical values not reported	- Exclusion: asthma, radiological pneumonia at the start of exacerbation, and/or a history of lung cancer, indication for non-invasive ventilation - Spirometrically gated FRC and TLC scans performed - HRCTs converted to 3D volumes in Mimics medical image processing software package - Note: patients in this study are likely a sub-population of the same clinical trial (NCT01684384) as in van Geffen, 2018.	**5**
**Kamel, 2013^25^**	105	unreported	- within 24h of admission	none	- Pulmonary embolism	- Pulmonary embolism in 30/105 (28.6%) of patients	- All patients hospitalized for suspected exacerbation	**0**
**Leong, 2017^12^**	64	- FEV_1_% = 48% ± 23%	- within 48h of hospital admission	n=17, 6-8 weeks post exacerbation	- Expiratory Central Airway Collapse	- Expiratory Central Airway Collapse (ECAC) prevalence was not a significant differentiator between stable COPD (14/40, 35%) and exacerbation (25/64, 39%, p = 0.835) - ECAC not found to be a significant biomarker of exacerbations (n=17): 53.2% ± 17.3% at exacerbation vs. 54.1 ± 18.9% at follow-up (p = 0.742) - ECAC was not a significant differentiator between exacerbation (53.8% ± 19.3%, n=64) and stable COPD (57.5 ± 19.8%, n=40, p = 0.355)	- Study focused on quantifying ECAC, which comprises Tracheal Obstruction (TO) and Excessive Dynamic Airway Collapse (EDAC) - Study also has a comparison group of n=40 stable COPD patients - ECAC defined as 50% tracheal area decrease - Exclusion criteria were known tracheal or laryngeal disease, a history of asthma, the inability to be recumbent for 10 min and known obstructive sleep apnoea	**3**
**Park, 2019^51^**	64	- FEV1% = 48.5% (32%-57%)	- within 72h of admission	none	- Infiltration - Nodules - Pericardial Effusion - Pleural effusion - Pulmonary artery diameter- Pulmonary congestion	- Pneumonic infiltration in 21/64 (33%) patients - Nodules in 2/64 (3%) patients - Pericardial effusion in 1/64 (2%) patients - Pleural effusion in 1/64 (2%) patients - Pulmonary artery enlargement in 1/64 (2%) patients - Pulmonary embolism in 1/64 (2%) patients - Oedema [assume Pulmonary congestion] in 1/64 (2%) patients	- Study running January 2010 to December 2012 - Study aimed to compare utility of CT at exacerbation in changing diagnosis or treatment - Excluded definite asthma, but included patients with bronchodilator-response positive - Exclude: any patientswho underwent a chest CT before the initial chest X-ray or 72hours after hospitalization - Contrast CT for 40 patients, non-contrast for 24 - Comparison group of 138 patients with no CT scan	**2**
**Rutschmann, 2006^26^**	123	- GOLD 2: 27 patients (22%) - GOLD 3: 61 patients (50%) - GOLD 4: 35 patients (28%)	unreported	none	- Pulmonary embolism	- Very low prevalence of pulmonary embolism in patients with exacerbation: PE detected in 4 (3.3%) of patients	- Consecutive inclusion of patients with confirmed exacerbations - Exclusion: renal failure (plasma creatinine >150 mmol/l), allergy to intravenous contrast, on long-term anticoagulation therapy at admission or in respiratory distress requiring intubation/non-invasive ventilation, obvious alternative cause of dyspnoea (lobar pneumonia, pneumothorax, pulmonary oedema and otherobvious causes)	**2**
**Shapira-Rootman, 2015^52^**	49	No stable state spirometry At exacerbation:post-BD FEV_1_% = 36%	unreported	none	- Atelectasis - Bronchiectasis - Consolidation - Emphysema - Fibrosis - Granuloma - Interstitial changes - Nodules - Peribronchial cuffing - Pleural effusion - Pulmonary artery diameter - Pulmonary embolism - Pulmonary congestion	- Atelectasis in 14/59 (23.7%) - Bronchiectasis in 5/49 (10.2%) - Emphysema in 23/49 (46.9%) - Consolidation in 7/49 (14.2%) - Granuloma in 4/49 (8.1%) - Interstitial changes in 8/49 (16.3%) - Peribronchial cuffing in 4/49 (8.1%) - Pleural effusion in 11/49 (22.4%) - Fibrosis (pleuropulmonary) in 14/49 (23.7%) - Pulmonary artery enlargement in 9/49 (18.3%) - Pulmonary embolism in 9/49 (18.3%) - Pulmonary nodules in 5/49 (10.2%) - Pulmonary oedema [grouped into pulmonary congestion] in 1/49 (2%) - Atelectasis and pulmonary artery enlargement in a significantly higher proportion of patients with PE than those without	- All patients hospitalized - Consecutive admission - Exclusion criteria: inability to consent, inability to perform spirometry, impaired renal function, contrast allergy, anticoagulant treatment, and known hypercoagulable state	**2**
**Tillie-Leblond, 2006^27^**	197	Available for 160/197 patients: FEV_1_% = 52% ± 19%	- within 48h of admission	none	- Pulmonary embolism	- Pulmonary embolism in 43/197 (22%) Location of PE (n (%) of all patients): - Central 20/197 (10.1%) - Segmental 21/197 (10.7%) - Isolated subsegmental 2/197 (1%) Note: values for PE prevalence on CTA reported (the study diagnosed 6 additional patients with PE based on lower extremity ultrasonography, leading to a total of 49 PE)	- ~70% patients were emergency referrals and 30% were inpatients who developed symptoms suggesting exacerbation - Only patients with ‘exacerbation of unknown origin’ included - unknown origin defined based on exclusion of purulence of sputum, history of a cold or sore throat, pneumothorax or iatrogenic intervention, or when there was a discrepancy between the clinical and radiologic features and hypoxemia severity - Excluded patients requiring mechanical ventilation	**2**
**Turk, 2017^28^**	36	Mean FEV_1_% = 46% ±15%	unreported	none	- Pulmonary embolism	- Pulmonary embolism in 13/36 (36.1%)	- All patients hospitalized - Retrospective study	**2**
**van Geffen, 2018^14^**	47	- GOLD 2: 19 patients - GOLD 3: 22 patients - GOLD 4: 6 patients	- within 5 days of the exacerbation start	All patients rescanned 42 days post exacerbation	- Airway resistance - Airway volume - Hyperinflation	At exacerbation vs. follow-up: - Hyperinflation - lobar volumes at FRC increased: 5.01±1.18 L vs. 4.75±1.10 L (p<0.01) - Airway Volume at TLC decreased: 54.79±16.05 mL vs. 56.49±16.32 mL (p=0.02) - Airway Resistance at FRC and TLC increased: - at FRC:0.11±0.13 kPa s/L vs. 0.06±0.08 kPa s/L (p=0.03); - at TLC: 0.04±0.03 kPa s/L vs. 0.04±0.02 kPa s/L (p=0.03)	- Only statistically significant changes in imaging features captured - Change in FEV1 correlates to change in specific airway volumes - Note: patients in this study are likely a sub-population of the same clinical trial (NCT01684384) as those in Hajian, 2018.	**5**
**Wang, 2016^13^**	79	- GOLD 1: 11 patients (14%) - GOLD 2: 25 patients (32%) - GOLD 3: 43 patients (54%)	unreported	none	- Area of small pulmonary vessels	Values based on a comparison between a group of exacerbating (n=79) patients with a group of stable COPD (n=74) patients: - Percentage of total lung area taken up by the cross-sectional area of pulmonary vessels less than 5mm^2^ (%CSA<5) significantly lower in the exacerbation group vs. stable COPD group: 0.41±0.13 vs.0.68±0.18 (p<0.001) - %CSA<5 found to be an indicator of exacerbation, cut off value was 0.56% (highest Youden index) with sensitivity and specificity of 0.836 and 0.731 - %CSA decrease associated with overall increase of COPD severity (for both the AE and stable COPD group)	- Both hospitalized and outpatients with exacerbation - Images analysed with ImageJ Version 1.48g - Exclusion: image noise that prevented image analysis (33 patients) and obvious severe lung lesions such as lung cancer, pulmonary tuberculosis and severe infection (19 patients)	**3**
**Wells, 2016^15^**	134	FEV_1_% = 47% ± 19%	unreported	- 12 months before exacerbation - A subset (n = 33) also had post exacerbation CT scan within 1-12 months	- Pulmonary Artery (PA) diameter - Pulmonary Artery to Aorta (PA/A) ratio	Significant changes in PA diameter and PA/A ratio during exacerbation as compared to baseline both before and after exacerbation. Reported as baseline-pre vs. exacerbation vs. baseline-post: - PA: 2.88 ± 0.52 cm vs. 3.07 ± 0.49 cm (p<0.001) vs. 2.85 ± 0.56 cm (p<0.001) - PA/A ratio: 0.91 ± 0.17 vs. 0.97 ± 0.15 (p<0.001) vs. PA/A = 0.91 ± 0.15 (p<0.001)	- Only hospitalized exacerbations included - Excluded if lung transplantation or if acute pulmonary embolism present on the exacerbation scan - A PA/A ratio > 1 found to predict cardiac injury and a more severe hospital course	**5**
**Coronary Angiography**								
**Pizarro, 2016^53^**	88	- GOLD A: 13 patients (15%) - GOLD B: 29 patients (33%) - GOLD C: 19 patients (22%) - GOLD D: 27 patients (31%)	- within 72h of admission	none	- Coronary artery diameter	Coronary artery diameter: - Ischaemic Heart Disease (IHD) in 59/88 (67%) - IHD defined as presence of a coronary stenosis >50% - In 34/88 (38.6%), revascularization was necessary - Single-, two-, and three-vessel disease in 26/88 (29.5%), 13/88 (14.8%), and 20/88 (22.7%) of patients - Right coronary artery preferentially affected and intervened (44.1% of patients requiring intervention)	- All patients hospitalized - Prospective study - Only exacerbation patients with elevated plasma troponin were included - Echocardiography was also performed	**2**
**Magnetic Resonance Imaging (MRI)**								
**Kirby, 2013^16^**	1	FEV_1_% = 41% - 47%	- within 8 days of admission	- 2 scans at 2.5y and 6m prior to AE - 1 scan 16m post AE	- MRI terms	Imaging biomarkers of ventilation:Timepoints in order 2.5y pre-AE, 6m pre-AE, 8d post-AE, 16m post-AE: - Ventilation defect percent (VDP) (%): 16, 29, 20, 14 - Apparent diffusion coefficient (ADC) (sq. cm/s): 0.34, 0.38, --, 0.34 - Antero-posterior ADC gradient slope reversed 6m pre-AE: “The elevated ADC in the posterior slices suggests dependent lung region gas trapping”. Gradient returned to baseline 16m post-AE.	- Hyperpolarized 3He imaging - Study on only 1 subject - Both ventilation and diffusion-weighted images acquired - Subject had reported deterioration in symptoms ~1m before exacerbation - No ADC measured during exacerbation (8 days after timepoint) due to technical difficulties	**2**
**Sergiacomi, 2014^17^**	15	All GOLD II - III	unreported	- Upon stabilization from exacerbation and before discharge from hospital	- MRI terms	- All biomarkers based on averaged values across the entire lung - Values presented for group of 6 patients exhibiting a change in parameters (remaining 9 exhibited no significant change). Reduction of pulmonary blood flow (PBF) at exacerbation vs. stable phase: - PBF (mL/100 mL of lung tissue/min): 64.3 ± 12.3 vs. 136.3 ± 14.4 (p<0.0001) Reduction of pulmonary blood volume (PBV) at exacerbation vs. stable phase: - PBV (mL/100 mL of lung tissue): 5 ±1 vs. 11.8 ± 4.2 (p=0.0059)Prolonging of the mean transit time (MTT) at exacerbation vs. stable phase:- MTT (s): 8.4 ± 1.5 vs. 4.6 ± 1 (p<0.0001)Prolonging of time to peak (TTP) at exacerbation vs. stable phase: - TTP (s): 4.9 ± 1.1 vs. 2.8 ± 0.7 (p=0.0034)	- All patients referred to the emergency department - Dynamic perfusion MRI (turbo field echo sequence) - All patients had exacerbation with hypercapnia and clinical signs of right heart failure- Inclusion criteria: aPaCO_2_ > 45 mmHg and respiratory acidosis (arterial blood pH < 7.35) at admission	**3**
**Ultrasound and Doppler Imaging**								
**Akcay, 2010^18^**	32	FEV_1_% = 65.9% ± 13.4%(post-treatment)	unreported	- 1 month after exacerbation	- USS terms	- Right ventricle (RV) systolic and diastolic function and left ventricle (LV) diastolic function impaired at exacerbation - Systolic tissue Doppler velocity (TSm) in the right ventricle RV increased at follow-up (after therapy) 13.7+2.4 vs. 14.4+2.4 cm/s (p = 0.027) - Diastolic RV and LV function improved at follow-up - Pulmonary artery pressures decreased at follow-up 34+5.2 vs. 28.2+4.7 mmHg (p<0.0001)- No change in systolic LV function	- Only patients without pulmonary hypertension included - Both US and Doppler performed; - 32 age- and sex-matched healthy control subjects also examined	**4**
**Antenora, 2017^36^**	41	unreported	- upon hospital admission	none	- Diaphragmatic disfunction	- Diaphragmatic disfunction defined as change in diaphragmatic thickness less than 20% during spontaneous breathing - Diaphragmatic disfunction in 10/41 (24.3%) (n=10) patients	- All patients had severe hypercapnic respiratory failure admitted to ICU, requiring non-invasive mechanical ventilation (NIV) - Trans-thoracic ultrasound performed - Prospective cohort study - Diaphragmatic disfunction associated with NIV failure and increased mortality at ICU	**1**
**Guo, 2018^54^**	655	unreported	- within 1 day of admission	none	- USS terms	- Left heart failure in 158/655 (24.1%) patients - diagnosed through echocardiography according to the American Society for Echocardiography guidelines: - systolic (108/158, 68.4%) - diastolic (50/158, 31.6%).	- Prospective cross-sectional observational study - Exclusion: renal dysfunction, acute myocardial infarction, cardiogenic shock, valvular heart disease, severe endocrine or hepatic dysfunction; also PE, pneumonia, pneumothorax, poor echogenicity - FEV1 provided during exacerbation- Also performed chest X-ray and CT, but not discussed- Left heart failure diagnosed through echocardiography based on consensus between 3 physicians	**2**
**Lepida, 2018^55^**	52	FEV1%: 44% ± 23%	just after stabilization (within the first 1 to 4 days afteradmission).	Separate cohort of 39 patients	- USS terms	- Echocardiography diagnosed pulmonary hypertension (PH) – according to the European Society of Cardiology guidelines - Possible or likely PH in 34 (65.4%) patients - Possible PH in 21 (40.4%) patients - Likely PH in 13 (25%) patients - Increased probability for likely/possible PH (echocardiographic evaluation) in exacerbation patients (p<0.0001)	- Study screened both exacerbation (n=52) and stable patients (n=39) - Chest X-ray also taken - Pulmonary hypertension defined as mean pulmonary artery pressure above 25 mmHg - Exclusion: “COPD patients with exacerbation withoutknown spirometry (n=7), with last spirometry more than 6 months before admission (n=4), sedatives in last 72 h before admission, cancer, stroke, other central nervous disorders unrelated to hypercapnic encephalopathy, major metabolic disorders, known thromboembolic disease, acute myocardial infarction within the last month and asthma”	**2**
**Lichtenstein, 1998^56^**	26 exacerbation patients (66 total dyspnoeic patients; remaining 40 with pulmonary oedema)	unreported	- No explicit reporting, but suggests soon after admission to ICU	none	- USS terms	Comet tail artefact defined as a differential imaging biomarker between exacerbation and pulmonary oedema: - Presence of comet tail artefact in 100% of cases with pulmonary oedema - Absence of comet tail artefact in 92% of cases with exacerbation (the 2 cases which had presence of comet tail artefact also had pneumonia) - The “Horizontal artefact” of the pleural line is visible in some exacerbation patients	- All patients hospitalized at ICU- Consecutively enrolled dyspnoeic patients - Also had 80 'normal' controls (no clinical or radiologic disorders) - Definition of comet tail artefact: “vertical hyperechogenic narrow-based repetition artefacts present bilaterally, either disseminated (defined as all over the anterolateral lung surface) or lateral (defined as limited to the lateral lung surface)”	**3**
**Lim, 2019^57^**	10	Mean FEV1/FVC, % =44.8% ± 12.6%	- Within 72 h after exacerbation	2 weeks after discharge	- Diaphragmatic dysfunction	- Right-side diaphragmatic thickening fraction [measure of Diaphragmatic dysfunction] significantly decreased at exacerbation as compared to stable state: 80.1 ± 104.9 mm vs. 159.5 ± 224.6 mm, p = 0.011 - Diaphragmatic thickening fraction defined as percent change in diaphragmatic thickness between end expiration and end inspiration	- Prospective study at one tertiaryhospital in Korea from January 2015 to March 2016 - Exclusion: presence of pulmonary diseases besides COPD, such as pleural effusion, pneumothorax, phrenic nerve palsy, and interstitial lung disease; medical history of chemical pleurodesis, neuromuscular disease, chest wall deformities; known pregnancy; and/or severeexacerbation of COPD requiring immediate endotracheal intubation	**3**
**Mantuani, 2016^58^**	57, Total dyspnoeic patients (not only exacerbation)	unreported	- Closely after admission	none	- Pericardial effusion - Pleural effusion - USS terms	Imaging features differentiating each of 3 main diagnoses were pre-agreed: Acute decompensated heart failure (ADHF): - Bilateral B-lines ("comet tail artefact”) (22/57, 38.5%) - Poor cardiac function (Decreased systolic LV function, 18/57, 31.6%) - Non-respirophasic inferior vena cava (IVC) COPD exacerbation: - Absence of B-lines - Normal or diminished cardiac function - A non-respirophasic or ﬂat IVC (19/57, 33.3%) Pneumonia: - Unilateral B-lines (13/57, 22.9%) - Consolidation - Hyperdynamic (15/57, 26.3%) or normal cardiac function - Non-plethoric IVC Study also reports the following US findings: - Pericardial effusion (2/57, 3.5%) - Pleural effusion (3/57, 5.3%) - Bilateral A-lines ("horizontal artefact”) (22/57, 38.5%) - Plethoric IVC (20/57, 35.1%) - Flat IVC (19/57, 33.3%)	- Prospective cohort study of patients presenting with acute dyspnoea due to ADHF, COPD exacerbations and pneumonia - Final diagnosis of exacerbation in 17/57, 30% of patients - Nonselective sample ("when investigator sonographers were present at the Emergency Department”) - Main aim was to differentiate ADHF, exacerbation, and pneumonia - “Unknown how many of all patients met our criteria for inclusion” – and did not record patients who met criteria when there was no investigator present - IVC classification: plethoric (<15% collapse), normal (15%-90% collapse), flat (>90% collapse)	**1**
**Marchioni, 2018^37^**	75	- All GOLD 4 - FEV1% = 47% (range 30–65%)	- on admission and before starting NIV	none	- Diaphragmatic dysfunction	- Ultrasound assessed diaphragmatic dysfunction, defined as a change in diaphragm thickness < 20% during tidal volume measured bilaterally at end inspiration and end expiration - Diaphragmatic dysfunction in 24/75 (32%) of patients	- Single centre prospective study - All patients had acute acidotic hypercapnic respiratory failure following AE, requiring NIV and admitted to the ICU - Patients with DD found to have a higher chance of NIV failure - Exclusion: “acute pulmonary oedema, coexistence of interstitial lung disease, history of neuromuscular disease, chest wall deformities, previously assessed diaphragmatic palsy, shock or severe hemodynamic instability, intracranial hypertension, known pregnancy, and/or need for immediate endotracheal intubation”	**2**
**Sriram, 2017^49^**	53	FEV_1_% = ~ 41% ± 18%	- within 24h of admission	none	- USS terms	Study explores the value of the B-lines test ("comet-tail artefact”) and relationship with B-type natriuretic peptides (BNPs) as markers of heart failure. Definition of a B-line positive test was the presence of at least 3 or more B-lines in at least one scan of each hemithorax - Positive B-lines test in 25/53 (47%) of patientsLung US B-lines test identifies subjects with a threshold level of BNP of >100 ng/L: - Positive predictive value 80% (59–93%) - Negative predictive value 64% (44–81%) - Positive likelihood ratio 3.1 (1.4–6.7) - Negative likelihood ratio 0.4 (0.3–0.7)	- All patients hospitalized - Convenience sample - Excluded patients with renal impairment, coexisting asthma and/or bronchiectasis, acute coronary syndrome or cardiac failure. - FEV_1_% value for 2 groups of patients presented in the paper: mean value approximated by author	**2**
**Zechner, 2010^59^**	1	- GOLD IV	- Nearly immediate (at the ambulance)	none	- USS terms	- Bilateral predominant A-Lines ("horizontal artefact”) at the anterior and lateral surface of the lung found indicative for exacerbation - Predominant B-Lines ("comet tail artefact”)across the anterior and lateral lung surface indicative of pulmonary oedema	- Case report - two cases examined to demonstrate utility of lung ultrasound in differentiating pulmonary oedema from COPD exacerbation and administering the appropriate treatment - US performed in ambulance - Correct diagnosis assessed by positive response to treatment	**2**
**Lung Ventilation Scintigraphy**								
**Cukic, 2014^60^**	20	No stable state spirometry.At exacerbation: - GOLD II: 14 patients (70%) - GOLD III: 4 patients (15%) - GOLD IV: 4 patients (15%)	unreported	none	- Emphysema	- Uses descriptive distribution patterns of radio-aerosol as markers of lung damage [assume emphysema] : - inhomogeneous deposition (ID) pattern - central deposition (CD) pattern - spotty deposition (SD) pattern - mixed deposition (MD) pattern - All patients found to have ID only or in combination with CD or with CD and SD	- Patients randomly selected from an intensive care unit - Also carried out body plethysmography - Paper aims to describe abnormal biomarkers in COPD, even though all patients were recruited during exacerbation - Individual patient data presented (GOLD stages calculated by author)	**1**
**Cunningham, 1981^31^**	30	unreported	unreported	follow-up imaging in 6 eCOPD patients at intervals of 8d - 23m	- V/Q mismatch	- Basal mismatched defects of ventilation at AE in 21/30 of patients - Basal mismatched defects of ventilation resolved in 6 eCOPD patients who had follow-up scans - Resolution of large matched defects (interpreted as obstructing bronchial lesions) in 2 of the 6 - In 2 eCOPD patients, unmatched defects of perfusion (interpreted as PEs) observed with partial resolution over 6w and 5m	- Study also enrolled 30 stable COPD patients for comparison - Results are often presented for all 60 patients so difficult to extract eCOPD from stable COPD - 23/60 patients had lung function tests; FEV1 for further 9 - Subjective assessment of scan abnormalities: for ventilation, lung divided into upper and lower zone and subjectively ranked from 0 (no activity) to 4 (normal activity) - Also performed chest X-ray, but findings not reported - Blind assessment of chest X-ray if mismatching was present	**2 **
**Erelel, 2002^29^**	56(only 8 had V/Q scan)	Mean FEV_1_/FVC% = 52% ± 13%)	unreported	none	- Pulmonary embolism	- Pulmonary embolism in 5/56 (8.9%)	- All patients hospitalized - Prospective study - V/Q scan performed on only 8 patients who had sudden chest pain with shortness of breath and/or hypocapnia on arterial blood gases and/or radiological suspicion of pulmonary embolus	**1**
**Selby, 1991^42^**	7	unreported	- within 36 h of admission	none	- V/Q mismatch	- Higher rate of neutrophil retention during first passage (22% ± 14.1%) in the lungs of exacerbation patients compared to stable COPD and elderly controls - Slower rate of neutrophil 'washout' from the lungs of exacerbation patients compared to stable COPD patients and elderly controls	- Neutrophil retention in 7 exacerbation patients compared to 14 stable COPD patients - Inverse correlation between neutrophil retention and emphysema extent - Performed gamma camera imaging with radio-labelled neutrophils - Performed CT on 9 stable COPD patients- Stable state FEV_1_ recorded but not presented	**1**
**Vibration Response Imaging (VRI)**								
**Bing, 2012^61^**	36	unreported	unreported	none	- VRI terms	VRI imaging biomarkers of exacerbation patients pre non-invasive ventilation: - low imaging synchronization between the left and right lungs- distinct image jumping- many dry and moist rales- unsmooth maximal energy frame (MEF) edges with turgor and defect	- Paper aimed to evaluate the initial therapeutic effect of NIV through VRI- Imaging was performed immediately after NIV initiation (soon after admission) - 4 VRI 'scans' performed at different time-points: pre-NIV, at 15min of NIV treatment, at 2h of NIV treatment, 15min after the end of NIV treatment - Included 39 healthy controls - Also included chest X-ray: abnormal in 26 patients (pleural effusion or consolidation, but no breakdown provided) with 10 cases of multi-lobular or multi-segmental disease.	**3**

**Notes:** This complete table contains detailed information extracted from each paper; Assumptions by the authors are presented in square brackets; Imaging biomarkers are presented as either quantitative measurements, or prevalence in a patient population.

**Abbreviations:** A, antibiotic; AE, acute exacerbation of COPD; AUC, area under the curve; BD, bronchodilator; CS, corticosteroid; CT, computed tomography; eCOPD, exacerbation of COPD; FEV_1_, forced expiratory volume in 1 second; FVC, forced vital capacity; GOLD I–IV, COPD severity assessed according to the Global Initiative for Chronic Obstructive Pulmonary Disease guidelines; HU, Hounsfield unit; ICU, intensive care unit; IQR, interquartile range; LAA%, percentage of low attenuation areas, a measure of emphysema; NIV, non-invasive mechanical ventilation; PE, pulmonary embolus; SD, standard deviation; US, ultrasound; V/Q, ventilation-perfusion; WA%, wall area percent – a measure of wall area thickness expressing the area taken up by the wall as a percentage of the total airway diameter.

In addition to the imaging biomarkers, further relevant information was captured from each article. Where described in the articles, we recorded COPD severity when stable: depending on the availability of this information, preference was given to GOLD stage, followed by forced expiratory volume in 1 second (FEV_1_) % predicted and finally FEV_1_/FVC (forced vital capacity). We recorded the timing of imaging to describe when imaging was performed in relation to the exacerbation time-course. Information on ascertainment of clinical diagnosis of exacerbation was collected and used for quality assessment (see below).

### Quality Assessment

Each study was assessed for quality using a 5-point grading system. We selected the Modified Newcastle-Ottawa Quality Assessment Scale (NOS) for Cohort Studies.^8^ It awards points to studies in three categories: Patient Selection (maximum 4 points), Comparability (maximum 2 points) and Outcome (maximum 3 points), allowing a maximum of 9 points for the highest quality studies. We modified the NOS to make it more applicable for the types of studies we were reporting – the original wording of each criterion from the NOS Scale, our modified decision rules in the context of imaging for COPD exacerbations and the maximum points awarded are described in Table S2. A confirmed clinical diagnosis of exacerbation was an inclusion criterion for our study, and the thoroughness of describing the criteria for confirming exacerbation contributed а point on our quality scale. For the modified criteria, the maximum score was 5 with a higher score representing higher quality. When synthesising evidence, we gave greatest weight to the studies of highest quality.

## Results

### Search Results

The search returned a total of 6,718 results. After removal of duplicates (n = 1,672), the final number of articles was 5,046. One additional study was identified soon after publication by speaking to a clinician involved in its design.

After screening the titles and abstracts of the 5,047 articles, we excluded 4,878 for reasons outlined in the PRISMA Flowchart (Figure 1).^3^ We excluded 118 further articles at full-text assessment, for the reasons also outlined in Figure 1. Fifty-one studies proceeded to full data extraction and qualitative synthesis.

### Imaging Biomarkers of Exacerbations of COPD

#### Included Studies

Table 1 presents the findings of the 51 studies included in the final analysis. In general, most studies discussed imaging biomarkers either as prevalence values of radiological findings in a population of subjects with an exacerbation of COPD, or as quantitative measurements of an imaging feature. A total of 10 of the 51 papers^9^–^18^ presented quantitative measurements of imaging features at exacerbation. One paper presented quantitative individual patient data, but no cohort averages,^19^ and the remaining 40 papers presented prevalence values for established radiological or other features. Most studies that presented quantitative values for imaging biomarkers performed consecutive imaging for the same subjects (at exacerbation compared to the same subjects at stable state as controls), whilst one study^13^ compared exacerbating subjects with a group of different stable-state COPD controls.

The identified imaging features are summarised in Table S1. Ten papers^20^–^29^ focused only on the prevalence of pulmonary embolism (PE) in patients hospitalized for suspected exacerbation of COPD. Most of these did not include details of any other imaging features and so are useful only in estimating the frequency of PE at exacerbation. Eleven serial imaging studies^10^–^12^,^14^–^19^,^30^,^31^ performed imaging at multiple time-points – usually at exacerbation and subsequent follow-up (assumed to represent recovery). The follow-up period for the second imaging time-point ranged from several days^17^ to 16 months after the initial exacerbation imaging.^16^ In all the studies, the end of exacerbation was clinically confirmed.

There were two studies which performed imaging both before and after exacerbation: Wells et al^15^ investigated changes in the pulmonary artery and aorta on CT, whilst Kirby et al^16^ studied ventilation and diffusion within the lung using hyperpolarized helium MRI, but only in a single subject.

**Figure 2:**
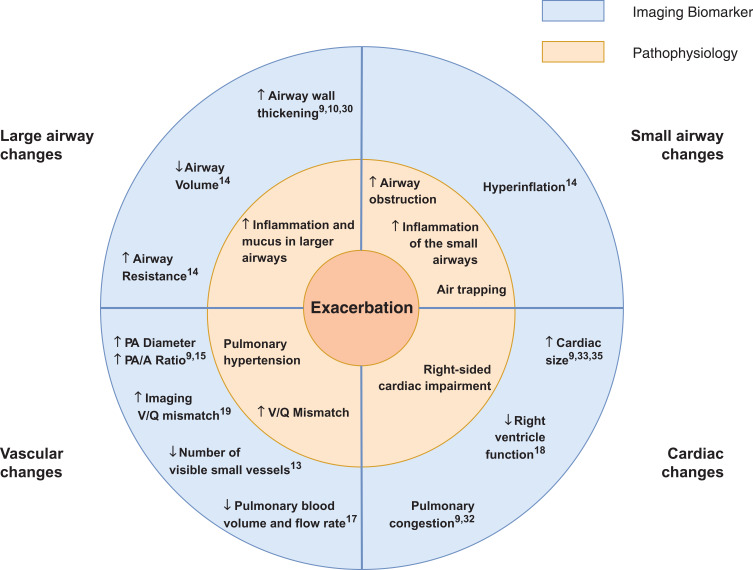
Summary: The Pathophysiology of a COPD Exacerbation in relation to Imaging Biomarkers. An exacerbation of COPD is associated with an increase in airway wall area and airway resistance, a decrease in airway calibre, hyperinflation, an increase in the diameter of the main pulmonary artery, a decrease in the number of visible small pulmonary vessels and cardiac enlargement. These features are suggestive of additional inflammation and mucus in the lumen of the airways, increased obstruction in the small airways and air trapping, pulmonary hypertension and right-sided cardiac impairment. **Abbreviations:** PA, pulmonary artery; PA/A ratio, pulmonary artery to aorta ratio; V/Q mismatch, ventilation perfusion mismatch; ↑, increased; ↓, decreased.

A total of 36 imaging features present during an exacerbation were reported by the included studies (Table S1). Conclusions, summarized in Figure 2, were primarily drawn from the 10 studies^10^–^15^,^17^–^19^,^30^ where a control group at stable state was present and the study scored three or more points on the Quality Assessment scale.

#### Chest CT

We identified 19 studies that reported chest computed tomography (CT) scanning during an exacerbation (Table 1). These studies provide the most detailed information on imaging biomarkers of exacerbations.

Prominent features of exacerbations include changes in bronchial wall geometry: Cheng et al^10^ found increased wall area percent (WA%) for the third generation airways and increased mean wall attenuation for the 3rd, 4th and 5th generation airways at exacerbation compared to follow-up. The increase in wall area as a percentage of the whole airway area may be an indicator of mucus or inflammatory infiltration within the lumen, while the increase in mean wall attenuation may represent inflammatory infiltration within the airway wall. Care must be taken with the latter parameter, however, since partial volume effect artefacts may lead to inaccurate measurements of attenuation of an airway wall with changing thickness. Bronchial wall geometry changes were also observed by Hackx et al,^30^ who noted bronchial wall thickening during an exacerbation, which decreased on follow-up. Van Geffen et al^14^ reported an increased imaging “airway resistance” both at total lung capacity (TLC) and functional residual capacity (FRC), derived by segmenting the airways and performing fluid dynamic modelling. This increased resistance could be explained by bronchoconstriction, airway wall changes and/or airway mucus plugging. Van Geffen et al additionally reported a decrease in total airway volume. Alotaibi et al^9^ also reported a high (67.5%) prevalence of airway thickening on CT, although this latter study only examined a single time-point scan.

Imaging features of the pulmonary vascular system also change at exacerbation. The study by Wells et al,^15^ which performed CT scans both up to 12 months before, at, and after exacerbation (the post-exacerbation scan was performed on a subsample of 33/134 patients), found statistically significant changes in the diameter of the main pulmonary artery (PA) and in the pulmonary artery to aorta (PA/A) ratio. The PA was enlarged during an exacerbation, leading to an increased PA/A ratio, whilst the PA diameter both before and after an exacerbation was reduced compared to at exacerbation. This distension of the pulmonary artery may suggest a degree of pulmonary arterial hypertension. Wang et al^13^ studied the number of small pulmonary blood vessels visible during exacerbation compared to stable COPD. The group segmented out blood vessels with a diameter 5–10mm, and those with a diameter <5mm, and defined metrics for the percentage of the total cross-sectional area of the lung taken up by each of the two vessel sizes – %CSA_<5_ and %CSA_5–10_. Interestingly, their results showed that %CSA_<5_ was significantly decreased at exacerbation, without a decrease in %CSA_5–10_. It should be noted, however, that the study did not scan patients at different time-points, but rather used two patient groups – one experiencing exacerbation and the other being stable COPD. The finding of fewer visible small blood vessels may represent vasoconstriction – the decrease in %CSA_<5_ may arise from smaller blood vessels in the lung narrowing during an exacerbation and falling below the resolution of CT. The authors also observed that reduction in %CSA was associated with increased COPD severity.

A further feature which changed between exacerbation and baseline was an increase in hyperinflation at FRC during exacerbation, quantified by van Geffen et al.^14^ A further study by the same group (on a subset of the same cohort), performed by Hajian et al^19^ also reported a significant improvement in the ventilation-perfusion ratio (V/Q) from exacerbation to baseline. The study quantified “imaging V/Q” by using lobar volume changes at TLC and FRC as a surrogate for ventilation and blood vessel density as a surrogate for perfusion. The V/Q improvement on recovery was mainly governed by improvement in the ventilation component, suggesting decreasing hyperinflation. There was no evidence of changes in emphysema extent (measured as LAA%), measured in two studies by Cheng et al.^10^,^11^ Lung hyperinflation is an imaging biomarker directly related to increased air trapping, which can be a consequence of increased dysfunction of the small airways or emphysema. The absence of significant changes in the extent of emphysema, though, suggests that an exacerbation does not directly lead to alveolar destruction.

Taken together, the CT studies indicate that exacerbations of COPD are events which likely manifest primarily in the airways and vasculature, with secondary changes such as hyperinflation in the parenchyma. Both smaller and larger vessels and airways are likely to be involved.

#### Chest X-Ray

Fourteen articles employed chest X-ray (Table 1). The imaging features visible on a chest radiograph during an exacerbation were all reported as prevalence values for radiographic features. Of the five highest-ranking studies:^9^,^32^-^35^ Alotaibi et al reported an increased cardiac size in 16.2%, Niksarlioglu et al in 19% and Shafuddin et al – in 25% of their populations respectively (however it is unlikely that this was an acute finding). Pleural effusion was reported in 11.6% of the population of Alotaibi et al and in 27% by Niksarlioglu et al Further prevalence findings in these two studies included pulmonary oedema in 15.5%,^9^ bronchiectasis in 31.7% and emphysema in 60.3%^33^ of patients – the latter two again are not likely to be acute. Johnson et al reported consolidation in 15% of patients and radiological features of congestive heart failure in 8%. Williams et al found new pneumonic infiltrates in 42.6% of their population and Niksarlioglu et al – in 54%.

#### MRI

We found two studies of chest MRI at exacerbation of COPD. Kirby et al^16^ performed hyperpolarized^3^ Helium imaging at four time-points to quantify ventilation defects at exacerbation, but their work can best be considered a pilot study since it only involved one subject. Sergiacomi et al^17^ used MRI to evaluate pulmonary perfusion and used an additional time-point early during recovery from exacerbation (before discharge from hospital) to compare imaging features. They found a reduction in pulmonary blood volume and pulmonary blood flow during an exacerbation, which is consistent with the CT studies described above. However, it should be noted that only six of 15 patients in the study exhibited changes.

#### Ultrasound

Ultrasound and Doppler imaging of the chest were performed in several studies which enrolled exacerbating patients. Most of these, however, scored low on our quality score and did not perform follow-up imaging, focusing on using imaging features such as the “comet-tail artefact” to differentiate between pulmonary oedema and exacerbation of COPD in patients presenting to the emergency room with deterioration in symptoms. The highest-rated ultrasound research was the work by Akcay et al^18^ which followed patients one month after the exacerbation and found that during an exacerbation patients experienced impaired right ventricular systolic and diastolic function, as well as impaired left ventricular diastolic function. The study specifically excluded patients known to have pulmonary hypertension. Cardiac impairment improved at follow-up, which suggests that an exacerbation might indeed be associated with a transient increase in pulmonary hypertension. Antenora and Marchioni reported on the prevalence of diaphragm dysfunction at exacerbation, and associations with hyperinflation.^36^,^37^

#### Other Imaging

Several papers featuring nuclear medicine techniques such as Lung Ventilation Scintigraphy, and more exotic techniques such as Vibration Response Imaging (VRI) were included in the final synthesis. In general, they scored low on our quality score, did not perform longitudinal imaging and did not provide useful imaging biomarkers of exacerbation.

## Discussion

We performed the first systematic review of imaging biomarkers at exacerbation of COPD. Such biomarkers might be useful in the objective diagnosis of exacerbation in the clinic and clinical trials, provide a mechanism for monitoring exacerbation treatment and recovery, and inform on the pathophysiological mechanisms of exacerbation.

Our results were strikingly consistent across studies, even though studies were heterogeneous in design. Most information was provided by CT, which consistently describes increases in wall area measures at the time of exacerbation, air trapping, changes in the pulmonary vasculature in keeping with vasoconstriction and suggestive of pulmonary hypertension, and the absence of changes in measures of emphysema. These changes are supported by an MR study demonstrating reduced pulmonary blood volume and flow,^17^ and echocardiography studies suggesting right heart dysfunction.^18^

Other CT studies, and those using chest X-ray, reported the prevalence of specific radiological features in patients with suspected exacerbations. These often demonstrated additional or alternative co-morbidities responsible for the deterioration in symptoms, which would thus meet the definition of exacerbation, but in which imaging changed the primary diagnosis away from COPD exacerbation. Some of these studies described multiple imaging features^9^,^30^ while others focused on identifying patients with a single possible alternative diagnosis such as PE. In both cases, the studies met our inclusion criteria for imaging during an exacerbation and consecutive enrolment of patients. Two large audits^38^,^39^ have demonstrated that the prevalence of consolidation on chest X-ray in COPD patients presenting with symptoms suggestive of exacerbation is 15–20% (and that this is associated with poorer outcomes). However, these scored low on our quality assessment as there was little available detail on patients or exacerbations. This emphasises the importance of developing imaging biomarkers of exacerbation: an imaging biomarker would enable a positive diagnosis of an exacerbation in clinical practice and research, rather than relying on the “clinical diagnosis of exclusion” model that is currently employed. CT would seem the best tool to achieve this. There were no positive diagnostic features on chest X-ray, whilst other bedside tests such as ultrasound have utility in the rapid exclusion of pulmonary oedema (“comet tail” artefacts), pneumothorax, pleural effusion and consolidation.

There are strengths to our review. Our comprehensive search strategy found 5,047 articles, of which 51 satisfied our inclusion criteria. While there is a significant body of literature on imaging features which correlate with, or sometimes predict exacerbations (for example the extent of emphysema^40^ and PA/A ratio^41^), few studies have scanned patients during an exacerbation and compared this with the baseline state in the same patients. A potential limitation of the literature is that most (but not all) studies were performed in hospitalised patients and therefore the events studied typically represent severe presentations (a combination of COPD severity and severity of the exacerbation insult). Patients with mild and moderate community-treated exacerbations are rarely imaged during an exacerbation and there is a real need for further research in this area. Another potential limitation is that patients at exacerbation may find it difficult to enforce the protocols needed for good scan quality (eg, breath-hold). However, none of the studies reported significant artefacts in imaging. Furthermore, modern CT scanners acquire images in seconds with impressive quality, such that imaging during an exacerbation becomes increasingly feasible.

**Figure 3:**
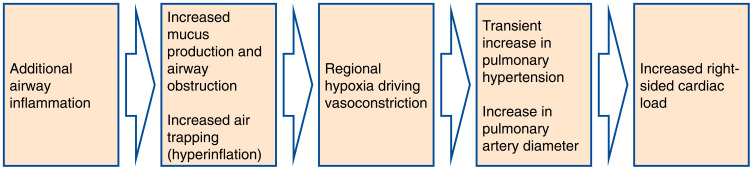
Hypothesis of the Pathophysiological Cascade Characteristic to an Exacerbation of COPD. Imaging features present during an exacerbation of COPD and their underlying pathophysiological processes motivate a hypothesis about the sequence of events in the lung which characterize an exacerbation. An exacerbation of COPD may be a cascade of events triggered by the onset of additional inflammation in the lumen of the airways, which leads to airway wall inflammation, bronchoconstriction and the production of excess mucus. This airway obstruction results in hyperinflation and the characteristic symptom of increased dyspnoea, and reactive vasoconstriction in small pulmonary vessels. In turn, this vasoconstriction leads to a transient increase in pulmonary arterial pressure, causing an enlargement of the pulmonary artery and right-sided cardiac dysfunction.

The included papers reported changes in different anatomical structures within the thorax and considered a variety of imaging biomarkers. While each of the articles focused on a particular aspect of exacerbation, systematically synthesizing the information has enabled us to generate a picture of the main features visible on medical imaging which characterize an exacerbation of COPD. These, together with their underlying pathophysiological processes, are summarised in Figure 2. One interpretation of the observed imaging characteristics of exacerbation, presented in Figure 3 is that an exacerbation of COPD represents a cascade of events triggered by the onset of additional inflammation in the airways, which leads to airway wall inflammation, bronchoconstriction and the production of excess mucus. This airway obstruction results in hyperinflation, and reactive vasoconstriction in small pulmonary vessels. In turn, the vasoconstriction leads to a transient increase in pulmonary arterial pressure, causing enlargement of the pulmonary artery and right-sided cardiac effects. There may be alternative explanations of the interrelatedness of the pathophysiological processes we have described.

For development as a tool in clinical trials, quantitative imaging measures are preferable to qualitative features. We did find quantitative measures of imaging features during exacerbation. Quantitative assessment of emphysema is already used in clinical practice to guide volume reduction interventions (but did not change at exacerbation). There are no existing quantitative imaging biomarkers of exacerbation or exacerbation recovery, and based on our work, plausible candidates might include assessment of airway wall area and volume, blood vessel calibre and volume, and gas trapping.

For use in the routine diagnosis and management of exacerbations, imaging biomarkers present an opportunity to achieve a quantitative, positive diagnosis of exacerbation, which could lead to reduction in misdiagnosis and an improved understanding of the pathophysiology of exacerbations. Quantitative exacerbation imaging metrics may also provide a tool to study exacerbation phenotypes which could further motivate development of novel treatments and permit a personalised medicine approach to exacerbation management.

A surprising finding was the absence of studies examining functional imaging such as positron-emission tomography. Specific cell-labelling techniques have demonstrated increased neutrophil retention within the lung at exacerbation,^42^ and slower rates of neutrophil wash-out compared to stable COPD and control subjects without COPD.

In conclusion, we have performed the first systematic review of imaging biomarkers at exacerbation of COPD. We find that an exacerbation is characterised by airway calibre and wall changes, hyperinflation, pulmonary vasoconstriction and pulmonary arterial hypertension. Exacerbation imaging biomarkers lay an important foundation for the objective diagnosis of exacerbation in the clinic and clinical trials, as well as for monitoring of exacerbation treatment and recovery.
